# Adipocyte‐specific ablation of plakoglobin in mice does not affect adiposity but results in sexual‐dimorphic effects on weight gain

**DOI:** 10.14814/phy2.70681

**Published:** 2025-12-26

**Authors:** Frederic Abou Azar, Alexis Vivoli, Arturo I. Machuca‐Parra, Frédéric Paré, Mete Civelek, Weinian Shou, Gareth E. Lim

**Affiliations:** ^1^ Department of Medicine Université de Montréal Montréal Quebec Canada; ^2^ Cardiometabolic Axis, Centre de Recherche du Centre Hospitalier de l'Université de Montréal (CRCHUM) Montréal Québec Canada; ^3^ Department of Anesthesiology and Perioperative Medicine University of California Los Angeles California USA; ^4^ Department of Human Genetics University of California Los Angeles California USA; ^5^ Division of Cardiology, Department of Medicine University of California Los Angeles California USA; ^6^ Institute for Precision Medicine, David Geffen School of Medicine University of California Los Angeles California USA; ^7^ Department of Pediatrics Herman B Wells Center for Pediatric Research, Indiana University School of Medicine Indianapolis Indiana USA

**Keywords:** adipocyte, beta‐catenin, body weight, obesity, plakoglobin

## Abstract

The main transcriptional coactivator of the WNT pathway, β‐catenin, is a well‐established regulator of adipogenesis and fat expansion, but our knowledge of how other members of the catenin family participate in adipogenesis remains incomplete. Previous studies have elucidated a role for the β‐catenin homolog, plakoglobin, in the regulation of adipogenesis in vitro, as its depletion impaired lipid accumulation. Moreover, plakoglobin overexpression in murine cardiomyocytes has been reported to enhance adipogenesis within the heart, further implicating its role as a key regulator of differentiation. In the present study, we investigated the adipocyte‐specific contributions of plakoglobin to adipogenesis and metabolism. Although deletion of plakoglobin in mature adipocytes had sex‐specific effects on body weight gain, whereby only chow‐fed knockout females weighed more than controls, no differences in adiposity or adipocyte size were observed. Moreover, no differences in glucose tolerance or insulin sensitivity were noted. Challenging mice with a high‐fat diet revealed diet‐induced metabolic disturbances only in male mice, but had no impact on adiposity or body weight. Together, our data demonstrate that plakoglobin in mature adipocytes is not required for adipogenesis or the expansion of adipose tissue mass.

## 1 INTRODUCTION

The obesity epidemic is a major problem affecting modern society. With rates of obesity increasing every year, more people are exposed to its risks and related comorbidities (Cooper et al., 2021; Yarnoz‐Esquiroz et al., 2022; Zimmet et al., 2001). Although highly effective treatments, such as incretin‐based therapies, have been developed, there still remains a limited number of overall therapies available to individuals living with obesity (Reid & Korner, 2022). Increasing efforts have been made to understand the underlying mechanisms of obesity and the expansion of adipose tissue mass, in the hopes of discovering novel therapeutic targets and expanding the choice of treatment options.

Increases in adipose tissue mass can be due to adipocyte hypertrophy or hyperplasia. It was previously reported that the number of adipocytes in humans remains stable throughout adulthood, with marginal changes in adipocyte turnover (Haczeyni et al., 2018; Horwitz & Birk, 2023; Spalding et al., 2008; White, 2023), but as individuals with obesity tend to have more adipocytes compared to leaner individuals, this suggests that obesity may also develop through adipocyte hyperplasia (Haczeyni et al., 2018; Horwitz & Birk, 2023; Spalding et al., 2008; White, 2023). Indeed, obesity‐induced hyperplasia has been confirmed through the AdipoChaser mouse model, as prolonged high‐fat diet feeding induced adipocyte hyperplasia during the expansion of gonadal adipose tissue (Wang et al., 2013). Furthermore, the increase in adipocyte hyperplasia has been shown to be facilitated via the upregulation of key adipogenic transcription factors, such as PPARγ2 (Jeffery et al., 2015). However, our knowledge of other factors that contribute to changes in adipose tissue expansion and adiposity remains incomplete.

The WNT pathway has previously been implicated in regulating the development of multiple metabolic tissues (Abou Azar & Lim, 2021). The main transcription co‐regulator, β‐catenin, has been shown to impair adipogenesis in vitro (Cawthorn et al., 2007; Choi et al., 2020; Li et al., 2007), and deletion of β‐catenin within mature adipocytes has been shown to protect mice from high‐fat diet‐induced obesity and reduce whole‐body adiposity (Bagchi et al., 2020; Chen et al., 2020). Plakoglobin is a close homolog of β‐catenin, and it has been shown to mediate WNT‐dependent signaling and actions, similar to β‐catenin, in various cell types (Lai et al., 2011; Lombardi et al., 2009; Maeda et al., 2004; Williams et al., 2000). Within cardiomyocytes, transgenic overexpression of plakoglobin led to fibrofatty replacement of cardiomyocytes, as its binding to TCF7L2 inhibited WNT‐dependent activity (Lombardi et al., 2009). Conversely, increased adipogenesis was not observed in mice with cardiomyocyte‐targeted plakoglobin deletion (Li et al., 2011). Plakoglobin has also been established as a regulator of insulin signaling and glucose uptake in mature adipocytes, with depletion of plakoglobin impairing both processes (Negoita et al., 2020). Despite these observations describing potential roles of plakoglobin in adipocytes, the precise in vivo contributions of plakoglobin, specifically in mature adipocytes, to adipocyte growth and function have yet to be explored in detail.

We previously defined a role for the scaffold protein 14‐3‐3ζ in regulating visceral adipogenesis (Lim et al., 2015), and one of the unique features of 14‐3‐3ζ is its ability to interact with diverse proteins harboring specific phospho‐motifs, resulting in large interactomes that can change in response to physiological and pathophysiological cues (Mugabo et al., 2018; Rial et al., 2023). Using mass spectrometry to assess how the 14‐3‐3ζ interactome is affected by high‐fat diet‐induced obesity, we were able to identify several novel proteins required for adipogenesis (Abou Azar et al., 2024). Plakoglobin belonged to a group of proteins that exhibited increased abundance within the 143‐3ζ interactome found in gonadal adipose tissue of obese mice, and through the use of murine and human pre‐adipocyte models, we were able to demonstrate important roles of plakoglobin in adipocyte differentiation and lipid storage in vitro (Abou Azar et al., 2024). However, whether these in in vitro functions apply to mature adipocytes in vivo is not known.

The primary objective of our study was to determine if plakoglobin is required for adipogenesis in vivo. Through selective deletion of plakoglobin in murine adipocytes we report that male and female mice fed chow or high‐fat diets displayed no differences in adiposity or adipocyte size. This suggests that plakoglobin is not required for adipogenesis and the regulation of fat mass in vivo. Interestingly, a sexual dimorphic role of plakoglobin in the regulation of body weight was observed whereby chow diet‐fed, female knockout mice gained more weight than controls. Under high‐fat diet feeding conditions, adipocyte‐specific deletion of plakoglobin did not affect weight gain in either sex, but it did affect insulin resistance in males. Altogether, these findings demonstrate that plakoglobin in adipocytes is not required for adipogenesis in vivo.

## 2 MATERIALS AND METHODS

### 2.1 Animal husbandry

*Adipoq*‐Cre mice (B6.FVB‐Tg(Adipoq‐cre)1Evdr/J, strain #028020, The Jackson Laboratory) were bred with mice harboring floxed alleles of *Jup*, the gene encoding plakoglobin, to generate adipocyte‐specific plakoglobin knockout (KO) mice (*Adipoq*‐Cre+; Jup^flox/flox^), and littermate controls (LC) (*Adipoq*‐Cre+; Jup^wt/wt^) were used in all studies. LoxP sites flanked exons 3‐5 (Li et al., 2011). All mice were on the C57BL6J background. Mice were placed on standard rodent chow (Teklad diet no. TD2918) or 60% high‐fat diet (HFD; Research Diets #12492). At the experimental endpoint, mice were euthanized by CO_2_ asphyxiation, followed by cervical dislocation. All procedures were approved by the Comité institutionnel de protection des animaux du CRCHUM (CIPA, protocol CM25001GLs) and performed in accordance with CIPA guidelines at the Université de Montréal Hospital Research Centre (CRCHUM).

### 2.2 Phenotypic correlations of adipose tissue derived from the hybrid mouse diversity panel (HMDP)

The HMDP represents one hundred inbred strains of 613 male and 783 female mice that were fed a standard lab diet (6% kcal from fat, Ralston Purina Company) for 8 weeks, followed by placement on a high‐fat /high‐sucrose diet (32% kcal from fat and 25% kcal from sucrose) for another 8 weeks (Parks et al., 2013, 2015). Total body fat and lean mass were measured and analyzed by MRI with Bruker Minispec and software from Eco Medical Systems (Houston, TX), respectively, every 2 weeks. After a 4‐hour fast, retro‐orbital blood was collected at endpoint from mice under isoflurane anesthesia. Plasma analysis for insulin, glucose, and triglycerides was determined (Castellani et al., 2008; Parks et al., 2013). HOMA‐IR was calculated by [([Glucose] × [Insulin])/405] (Parks et al., 2015). Flash‐frozen perigonadal adipose tissue samples were processed for gene expression analysis with an Affymetrix HT_MG430A array. The WGCNA package in R was used to determine gene expression‐phenotype correlations (biweight midcorrelation), in addition to significance (bicor function) (Langfelder & Horvath, 2012). In the present study, the HMDP dataset was reanalyzed to explore correlations between *Jup* (1426873_s_at) or *Ctnnb1* (1450008_a_at) and body fat or HOMA‐IR.

### 2.3 Metabolic phenotyping

Glucose and insulin tolerance tests and were performed on fasted mice, using D‐glucose via intraperitoneal administration (2 g/kg body wt; #G8270‐1KG, VWR, Solon, OH), or Human R insulin (0.5 U/kg body wt; Eli Lilly, Toronto, ON, Canada), respectively. Mice were fasted for 6‐ or 4‐hours, prior to the administration of glucose or insulin, respectively. Blood glucose was measured via tail blood using a Contour Next EZ glucose meter (Ascencia Diabetes Care, Basel, Switzerland). Body composition was determined with an EchoMRI‐100 Body Composition Analyzer (version 2008.01.18, EchoMRI LLC). Lipolysis was stimulated via intraperitoneal injections of isoproterenol (10 mg/kg; #I6504‐100MG, Sigma Aldrich, Saint Louis, Missouri, USA), and blood from the tail vein was collected after 20 min (Oppong et al., 2020). Free fatty acid concentrations were measured via the nonesterified fatty acid kit (NEFA; #633‐52001, WAKO Diagnostics, Osaka, Japan). Perigonadal and inguinal fat pads were isolated following euthanasia, and they were either flash frozen or stored in 4% paraformaldehyde for histological analyses or subjected to ex vivo lipolysis experiments, as previously described (Oppong et al., 2020; Rial et al., 2025).

### 2.4 RNA isolation and qPCR

RNA was isolated from tissue samples using Trizol reagent (#52202, ThermoFisher Scientific). cDNA was generated using the High‐Capacity cDNA Reverse Transcription Kit (#4368814, ThermoFisher Scientific, Waltham, MA). The QuantStudio 6‐flex Real‐time PCR System (ThermoFisher Scientific) was used to measure mRNA using SYBR Green chemistry (#A25742, ThermoFisher) (Abou Azar et al., 2024; Diallo et al., 2020; Mugabo et al., 2018, 2022; Oppong et al., 2020). Gene expression levels were quantified using the comparative Ct (ΔΔCt) method. All data were normalized to *Hprt*. Primers were obtained from Integrated DNA Technologies (IDT, Coralville, IA), and primer sequences are listed in Table 1.

**TABLE 1:** Sequences of primers used in this study.

*Ywhaz*	Reverse	CTT TCT GGT TGC GAA GCA TTG GG
	Forward	CAG AAG ACG GAA GGT GVT GAG A
*Ctnnb1*	Reverse	TGG GAG AAT AAA GCA ACT GCA CA
	Forward	GTT AAA CTC CTG CAC CCA CCA TAC
*Jup*	Reverse	GAC CAG GAT CTT CAG CAC ACT CT
	Forward	CCT GTG GAC TCT GCG CAA T
*Hprt*	Reverse	CCT GGT TCA TCA TCG CTA ATC
	Forward	TCC TCC TCA GAC CGC TTT T
*Pparg* E1	Reverse	GGC CAG AAT GGC ATC TCT CTG TGT GTC AA
*Pparg2* Fw	Forward	GTT ATG GGT GAA ACT CTG GGA GT
*Fabp4*	Reverse	AGTACTCTCTGACCGGATGG
	Forward	GGAAGCTTGTCTCCAGTGA
*Lpl*	Reverse	GTGACCGATTTCATCAAGTTTGGAG
	Forward	GACGGACACAAAGTTAGCACCAC
*Adipoq*	Reverse	CCCAAGGGAACTTGTGCAGGTTGGATG
	Forward	GTTGGTATCATGGTAGAGAAGAAAGCC
*Pdgfra*	Reverse	TCCTTCTACCACCTCAGCGAG
	Forward	CCGGATGGTCACTCTTTAGGAAG
*Cebpd*	Reverse	ACG ACG AGA GCG CCA TC
	Forward	TCGCCGTCGCCCCAGTC
*Cepbb*	Reverse	GCAAGAGCCGCGACAAG
	Forward	GGCTCGGGCAGCTGCTT
*Myog*	Reverse	CTGAAGGTGGACAGGAAGG
	Forward	GACCTGATGGAGCTGTATGAG
*Il6*	Reverse	GTCCTTAGCCACTCCTTCTG
	Forward	CAAAGCCAGAGTCCTTCAGAG
*Fndc5*	Reverse	GAAGGTCCTCTCGCATTCTC
	Forward	CAACGAGCCCAATAACAACA
*Fstn*	Reverse	TTATGATGGG CACTGCAAAGAA
	Forward	ACTGCCTTTAGAGAACCAGCC
*Mstn*	Reverse	AGTGGATCTAAATGAGGGCAGT
	Forward	GTTTCCAGGCGCAGCTTAC
*Hsl*	Reverse	GAATCGGCCACCGGTAAAGAG
	Forward	ACCGAGACAGGCCTCAGTGTG
*Atgl*	Reverse	AACACCAGCATCCAGTTCAA
	Forward	GGTTCAGTAGGCCATTCCTC

### 2.5 Histology

Following the sacrificing of mice, adipose tissues were harvested and stored in 4% paraformaldehyde (#158127, Sigma‐Aldrich) for 7 days at 4°C, before being stored in 70% ethanol. Tissues were then embedded in paraffin and sectioned at 5 μm thickness. Hematoxylin (#H3136, Sigma Aldrich) and eosin (#318906, Sigma Aldrich) staining was performed on tissue sections. Images of adipocytes were taken at a magnification of 20X (Leitz Diaplan microscope, Q‐Capture Pro 7.0; Teledyne QImaging, Surrey, Canada). Quantification of adipocyte size distribution was performed using ImageJ (v1.54g), following previously established protocols (Chen & Farese Jr., 2002).

### 2.6 Immunoblotting

Gonadal adipose tissues were homogenized in RIPA buffer (0.9% NaCl, 1% v/v Triton X‐100, 0.5% sodium deoxycholate, 0.1% SDS, and 0.6% Tris base), supplemented with a protease inhibitor (Sigma‐Aldrich). Following centrifugation, tissue lysates were collected and centrifuged, followed by protein quantification by the Bradford method. Equal amounts of protein were resolved by SDS‐PAGE, transferred to PVDF membranes (Bio‐Rad Transblot Turbo, Bio‐Rad, Hercules CA), and incubated overnight with antibodies against ATGL (#2439, Cell Signaling Technology, Danvers MA), HSL (#4107, Cell Signaling Technology), and β‐actin (#4967, Cell Signaling Technology). Following successive washes, membranes were incubated with secondary antibodies conjugated to HRP (anti‐rabbit IgG, #7074 or anti‐mouse IgG, #7076, Cell Signaling Technology), incubated with ECL, and imaged on a ThermoFisher Scientific iBright 1500CL. Densitometric analysis was performed using iBrightAnalysis Software (Ver 4.0).

### 2.7 Statistics

Data are expressed as mean ± standard error (SEM) or as mean ± standard deviation (SD), when appropriate. For experiments involving live animals, each group, LC or KO, included 5–6 biological replicates. Analysis of tissues for histology, gene expression, or protein abundance, each experimental condition includes 3–6 biological replicates. Statistical analyses were performed with GraphPad Prism 8 (GraphPad Software, San Diego, California, USA) by using Student's *t*‐test or one‐way ANOVA, where appropriate.

## 3 RESULTS

### 3.1 Expression of Ctnnb1 and Jup are associated with differences in body fat and insulin sensitivity

**FIGURE 1:**
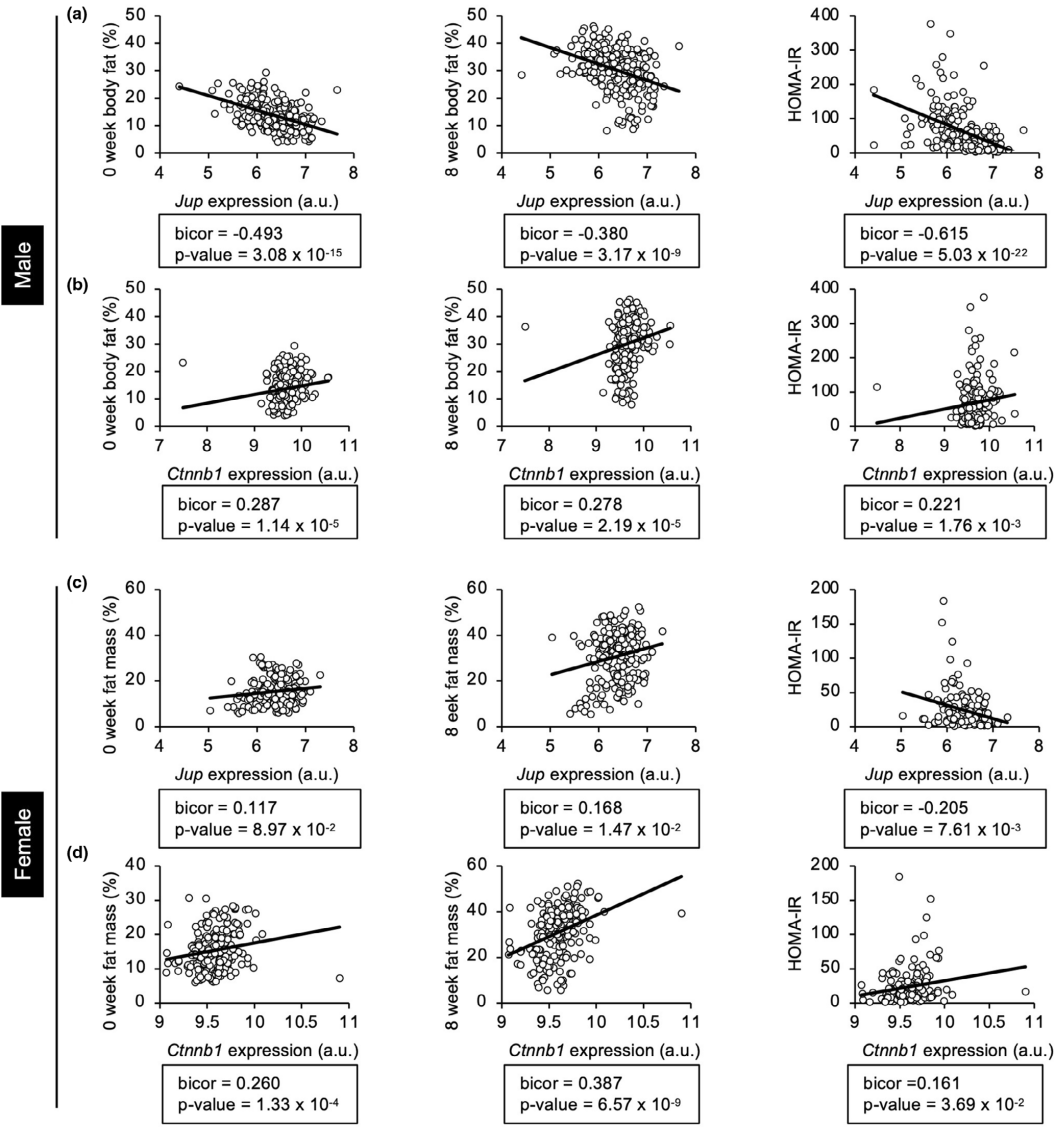
The relationship between *Jup* expression and %body fat differs between male and female mice. Correlations between *Jup* or *Ctnnb1* expression in perigonadal adipose with % fat mass, before and after 8 weeks of high‐fat, high‐sucrose feeding or HOMA‐IR from 100 inbred strains of male (a, b) and female (c, d) mice were determined by reanalyzing data from the Hybrid Mouse Diversity Panel (HMDP) (Parks et al., 2013, 2015).

To initially assess the relationship between plakoglobin and adiposity, we used the Hybrid Mouse Diversity Panel (HMDP) to determine how natural variations in the expression of *Jup*, the gene encoding plakoglobin, within adipose tissue correlates with fat mass or HOMA‐IR (Parks et al., 2013, 2015). The HMDP also allowed for comparisons to *Ctnnb1*, the gene encoding β‐catenin. In male and female mice, a sex‐dependent relationship between the degree of *Jup* expression in gonadal adipose tissue and %body fat was observed, where expression of *Jup* was negatively correlated to %body fat in chow‐ and HFHS diet‐fed male mice (Figure 1a). In contrast, %body fat of female mice fed either diet was positively correlated to *Jup* expression (Figure 1c). Although plakoglobin and β‐catenin share a higher degree of homology and share overlapping functions (Aktary & Pasdar, 2012; Ben‐Ze'ev & Geiger, 1998; Zhurinsky et al., 2000), *Ctnnb1* expression was inversely correlated to *Jup* with respect to %body in male mice fed chow or HFHS diets (Figure 1b,d). Insulin sensitivity, as measured by HOMA‐IR, was not influenced by sex, but instead was inversely correlated to *Jup* expression (Figure 1).

### 3.2 Sex‐specific differences in body weight gain following plakoglobin deletion in mature adipocytes

**FIGURE 2:**
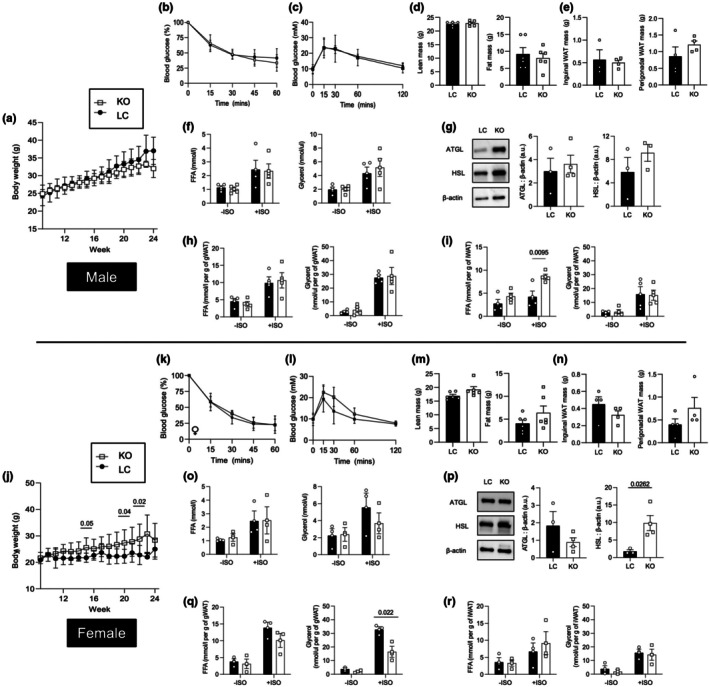
Ablation of plakoglobin led to weight gain in female mice. (a, j) Body weights of male (a) and female (j) mice over 6 months (*n* = 6 mice per group). (b, c and k, l) Intraperitoneal insulin (b and k; 0.5 U/kg body weight) and glucose (c and l; 2 g/kg body weight) tolerance tests at 6 months of age (*n* = 5–6 mice per group) (d, m) Lean and fat mass, as measured by ECHO‐MRI, at 6 months of age in knockouts (KO) and littermate controls (LC) (*n* = 5–6 mice per group). (e, n) Mass of inguinal and perigonadal fat pads, representing subcutaneous and visceral adipose tissue, respectively (*n* = 4 mice per group). (f, o) Measurement of plasma‐free fatty acids (FFA) and glycerol at 0 and 20 min (T0 and T20, respectively) following isoproterenol injection (*n* = 4 mice per group). (g, p) Protein abundance of HSL and ATGL from gonadal adipose tissues for male (g) and female (p) KO and LC mice (*n* = 3–4 mice per group). (h, i, q, r) Gonadal (h, q) and inguinal (i, r) adipose tissue explants from male (h, i) and female (q, r) mice were treated with 1 μM isoproterenol, followed by measurements of FFAs or glycerol in the supernatant (*n* = 4–5 biological replicates per group). Statistical significance is indicated by numerical *p* values, and significance was determined by unpaired *t*‐tests or one‐way ANOVA. Graphs for body weight, IPGTT and IPITT represented by mean ± SD. All other bar graphs represent mean ± SEM.

Although the HMDP suggests that levels of *Jup* expression correlate with %body fat in a sex‐dependent manner (Figure 1a,c), it was not clear what cell type within adipose tissue accounts for this relationship. We previously reported that plakoglobin was required for adipogenesis in 3T3‐L1 pre‐adipocytes and lipid accumulation in vitro (Abou Azar et al., 2024), and in order to elucidate the adipocyte‐specific role of plakoglobin in vivo, we generated mice with mature adipocyte‐specific deletion of *Jup*. After 24 weeks of chow diet, no discernible changes in body weight, fat mass, or lean mass were observed in male mice (Figure 2a,d,e). The lack of effects on body weight or fat mass was surprising given the negative correlation between *Jup* expression and %body fat from the HMDP data (Figure 1a,c). Glucose tolerance and insulin sensitivity of male KO mice were similar to littermate controls (Figure 2b,c). Female KO mice exhibited an increase in weight gain on a chow diet (Figure 2j), but this increase was not due to changes in lean mass, fat mass, or fat pad weights (Figure 2m,n). Glucose tolerance and insulin sensitivity were unaffected by plakoglobin deletion in female mice (Figure 2k,l).

Isoproterenol‐induced lipolysis was unaffected by adipocyte‐specific plakoglobin deletion in male or female mice (Figure 2f,o), despite observing differences in HSL protein abundance (Figure 2g,p). Ex vivo analysis of isoproterenol‐mediated lipolysis revealed no depot‐specific differences in free fatty acid or glycerol release from inguinal or gonadal adipose tissues from male and female mice, respectively (Figure 2h,i,q,r). Altogether, these findings demonstrate that plakoglobin in adipocytes likely does not influence adiposity or whole‐body metabolism.

### 3.3 Deletion of Jup does not alter adipocyte size

**FIGURE 3:**
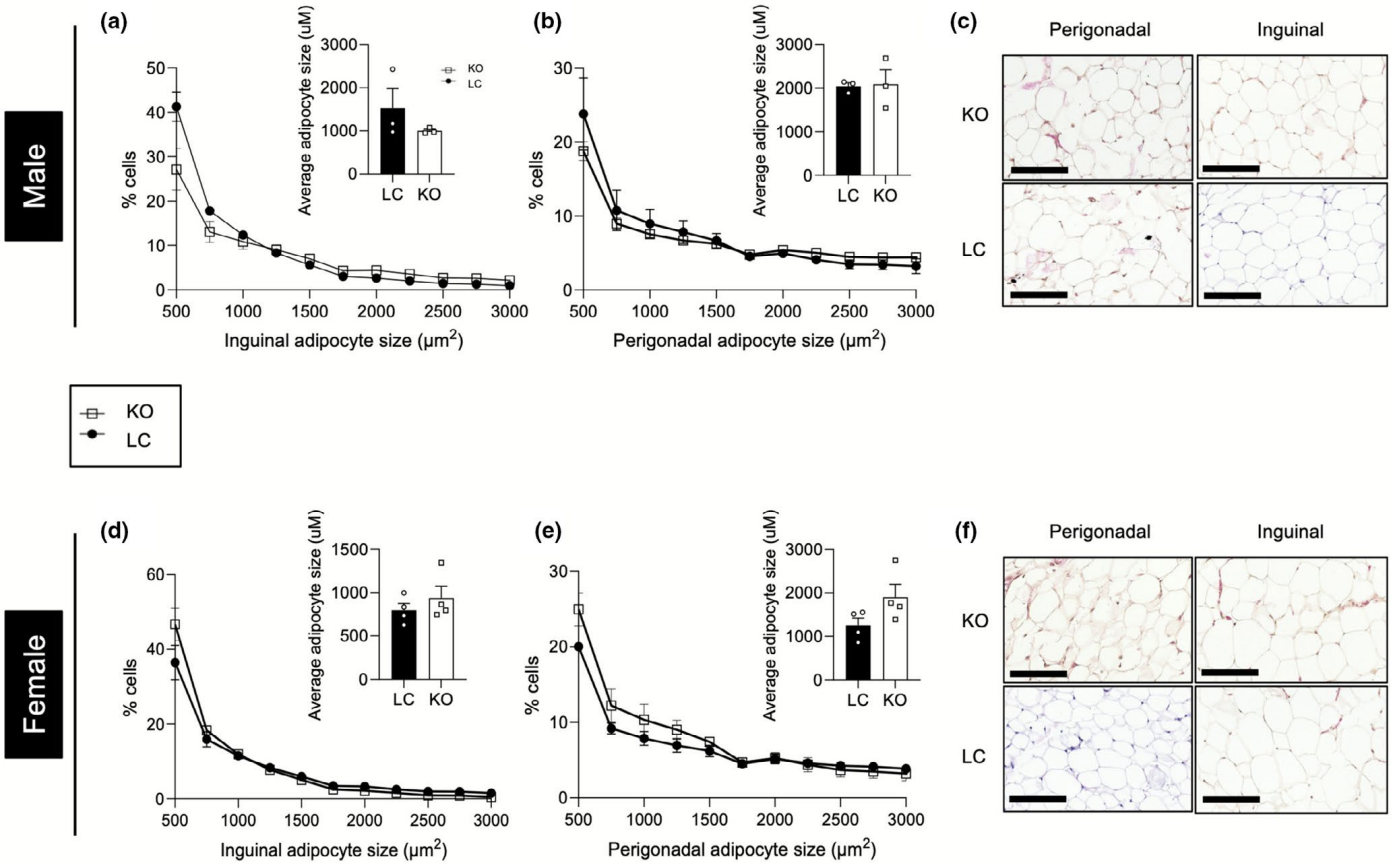
Loss of plakoglobin does not affect adipose tissue remodeling. (a, b) Size distribution of adipocytes with corresponding analysis of white adipocyte average size from inguinal and perigonadal fat pads from 24 week old LC and KO male mice (*n* = 3 mice per group). (c) Representative images of male inguinal and perigonadal white adipocytes (scale bar = 300 μM). (d, e) Size distribution of adipocytes with corresponding analysis of white adipocyte average size from inguinal and perigonadal fat pads from 24‐week old LC and KO female mice (*n* = 4 mice per group). (f) Representative images of female inguinal and perigonadal white adipocytes (scale bar = 300 μM). Bar graphs represent mean ± SEM.

Adipocyte expansion can occur via hypertrophy or hyperplasia (Haczeyni et al., 2018; Horwitz & Birk, 2023; Spalding et al., 2008; White, 2023), and to investigate any potential differences in adipocyte size and number, morphometric analyses were performed on perigonadal and inguinal fat pads. Histological analyses confirmed our observation of no differences in adiposity, as the overall size and distribution of inguinal and perigonadal adipocytes from male KO mice were not significantly different (Figure 3a–c). Similarly, no significant differences in adipocyte size or distribution were observed between female KO mice and littermate controls (Figure 3d–f).

**FIGURE 4:**
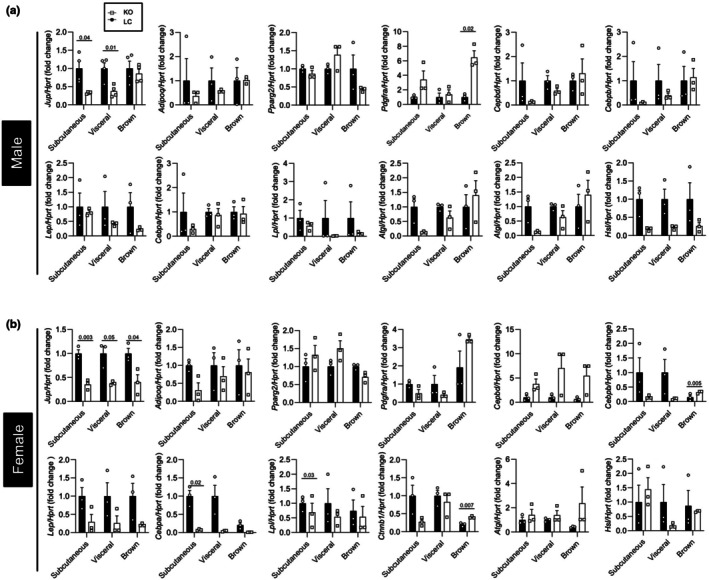
Adipogenic gene expression is not disrupted by *Jup* knockout. (a) Changes in mRNA expression of selected adipocyte‐related genes in male subcutaneous, visceral and brown fat pads (*n* = 3–4 mice per group). (b) Changes in mRNA expression of selected adipocyte‐related genes in female subcutaneous, visceral, and brown fat pads (*n* = 3 mice per group). Statistical significance is indicated by numerical *p* values, and significance was determined by unpaired *t*‐tests. Bar graphs represent mean ± SEM.

To further confirm that plakoglobin expressed in adipocytes was not necessary for adipogenesis, we measured the expression of genes associated with adipocyte maturity or function. As expected, *Jup* mRNA was significantly decreased in adipose tissues from male and female mice (Figure 4), and the majority of genes associated with adipocyte maturity or function were not significantly different between KO and littermate control male or female mice (Figure 4).

### 3.4 Plakoglobin in adipocytes is dispensable for high‐fat diet‐induced weight gain

**FIGURE 5:**
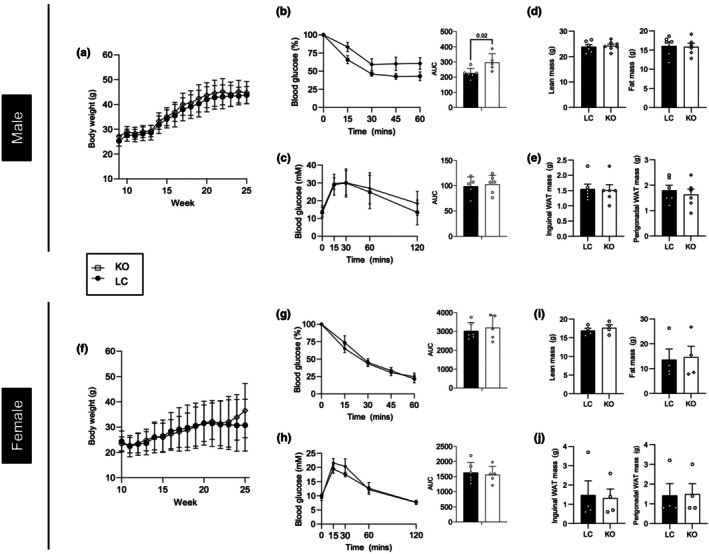
Diet‐induced obesity affects insulin sensitivity in adipocyte‐specific *Jup* knockout male mice. (a, f) Body weight of male (a) and female (f) mice over 6 months (*n* = 4–6 mice per group). (b, c and g, h) Intraperitoneal insulin (b and g; 0.5 U/kg body weight) and glucose (c and h; 2 g/kg body weight) tolerance tests at 6 months of age (*n* = 4–6 mice per group). (d, i) Lean and fat mass, as measured by ECHO‐MRI, at 6 months of age in knockouts (KO) and littermate controls (LC) (*n* = 5–6 mice per group). (e, j) Mass of inguinal and perigonadal fat pads, representing subcutaneous and visceral adipose tissue, respectively (*n* = 4–6 mice per group). Statistical significance is indicated by numerical *p* values; significance was determined by unpaired *t*‐tests. Graphs for body weight, IPGTT, and IPITT are represented by mean ± SD. All other bar graphs represent mean ± SEM.

**FIGURE 6:**
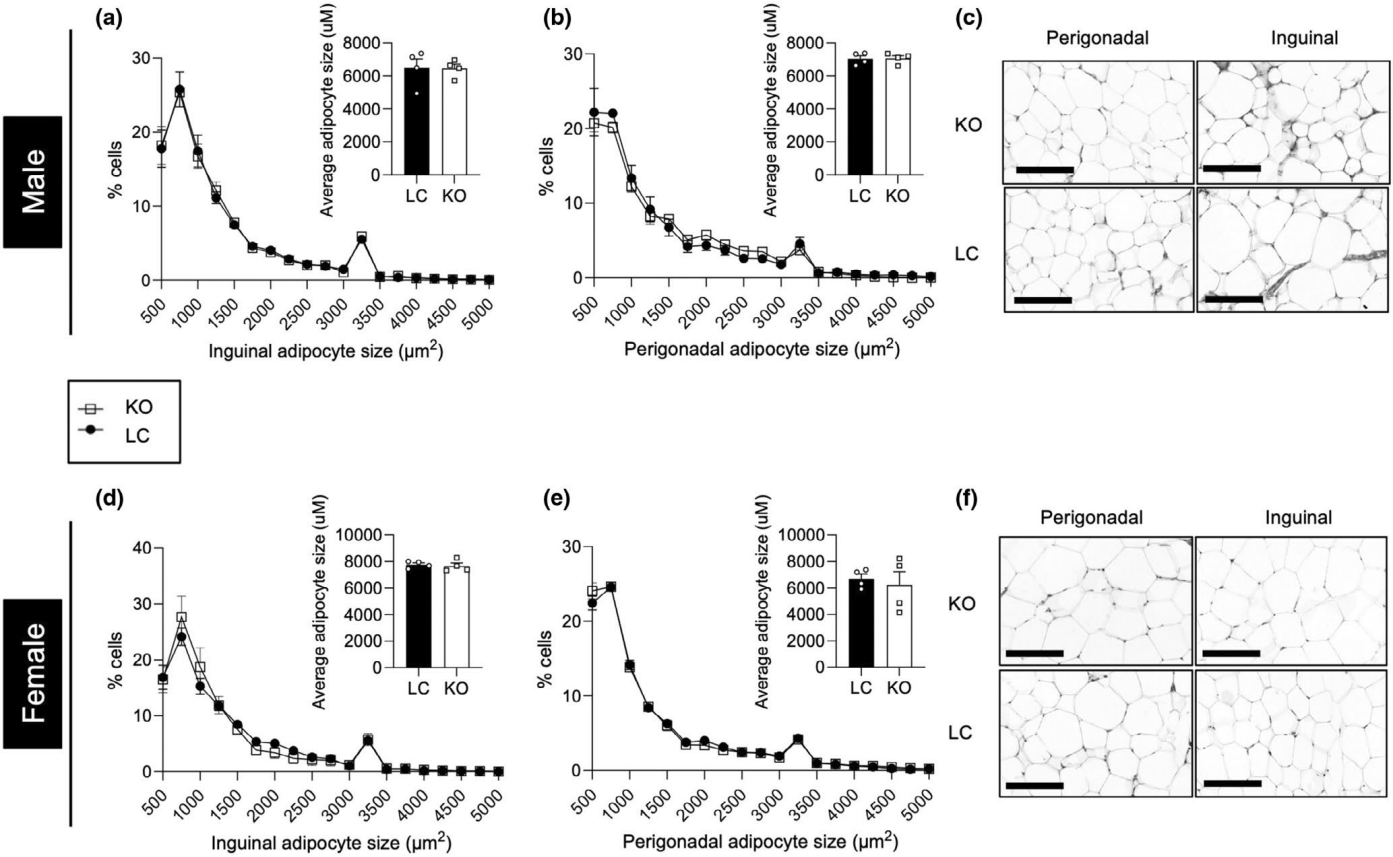
High‐fat diet does not affect adipocyte size. (a, b) Size distribution of adipocytes with corresponding analysis of white adipocyte average size from inguinal and perigonadal fat pads from LC and KO male mice following high‐fat diet (*n* = 4 mice per group). (c) Representative images of male inguinal and perigonadal white adipocytes (scale bar = 300 μM). (d, e) Size distribution of adipocytes with corresponding analysis of white adipocyte average size from inguinal and perigonadal fat pads from LC and KO female mice fed a high‐fat diet (*n* = 4 mice per group). (f) Representative images of female inguinal and perigonadal white adipocytes (scale bar = 300 μM). Bar graphs represent mean ± SEM.

**FIGURE 7:**
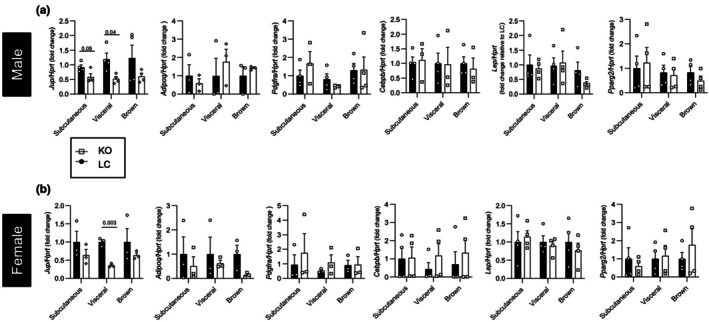
Adipogenic genes are unaffected by high‐fat diet. Changes in mRNA expression of selected adipocyte‐related genes in male (a) and female (b) subcutaneous, visceral, and brown fat pads (*n* = 3–4 mice per group). Statistical significance is indicated by numerical *p* values, and significance was determined by unpaired *t*‐tests. Bar graphs represent mean ± SEM.

Extensive studies have shown the WNT pathway to be involved in adipocyte metabolism during periods of nutrient excess (Bagchi et al., 2020; Geoghegan et al., 2019; Longo et al., 2004; Nguyen‐Tu et al., 2021; Wang et al., 2025). Although the role of plakoglobin in the Wnt pathway has been established, its contributions to adipocyte maturation and metabolism have not been extensively studied (Abou Azar et al., 2024; Aktary & Pasdar, 2012; Bierkamp et al., 1999; Fukunaga et al., 2005; Garcia‐Gras et al., 2006; Maeda et al., 2004; Negoita et al., 2020). We previously reported that plakoglobin abundance was increased in gonadal adipose tissue of mice fed a 60% HFD, which suggests that it could be involved in the expansion of adipose tissue during nutrient excess (Abou Azar et al., 2024). Thus, to evaluate the impact of plakoglobin ablation in adipocytes on the development of HFD‐induced obesity, KO mice or littermate controls were challenged with a 60% HFD for 12 weeks. No differences in weight gain were observed in male or female KO mice (Figure 5a,f). While glucose tolerance was unaltered in male mice, decreased insulin sensitivity was observed (Figure 5b,c). Female mice exhibited no differences in systemic metabolism (Figure 5g,h). Fat mass and lean mass remained unchanged in both sexes (Figure 5d,i), and inguinal and perigonadal adipose depots did not display any changes in mass (Figure 5e,j). Histological analyses revealed no differences in perigonadal or inguinal adipocyte size and density distribution for either sex under high‐fat diet feeding (Figure 6). Similar to chow‐fed animals, mRNA levels of genes associated with adipocyte maturity were unaffected in both sexes (Figures 4 and 7).

### 3.5 Plakoglobin deletion does not affect the expression of myogenic genes in skeletal muscle

**FIGURE 8:**
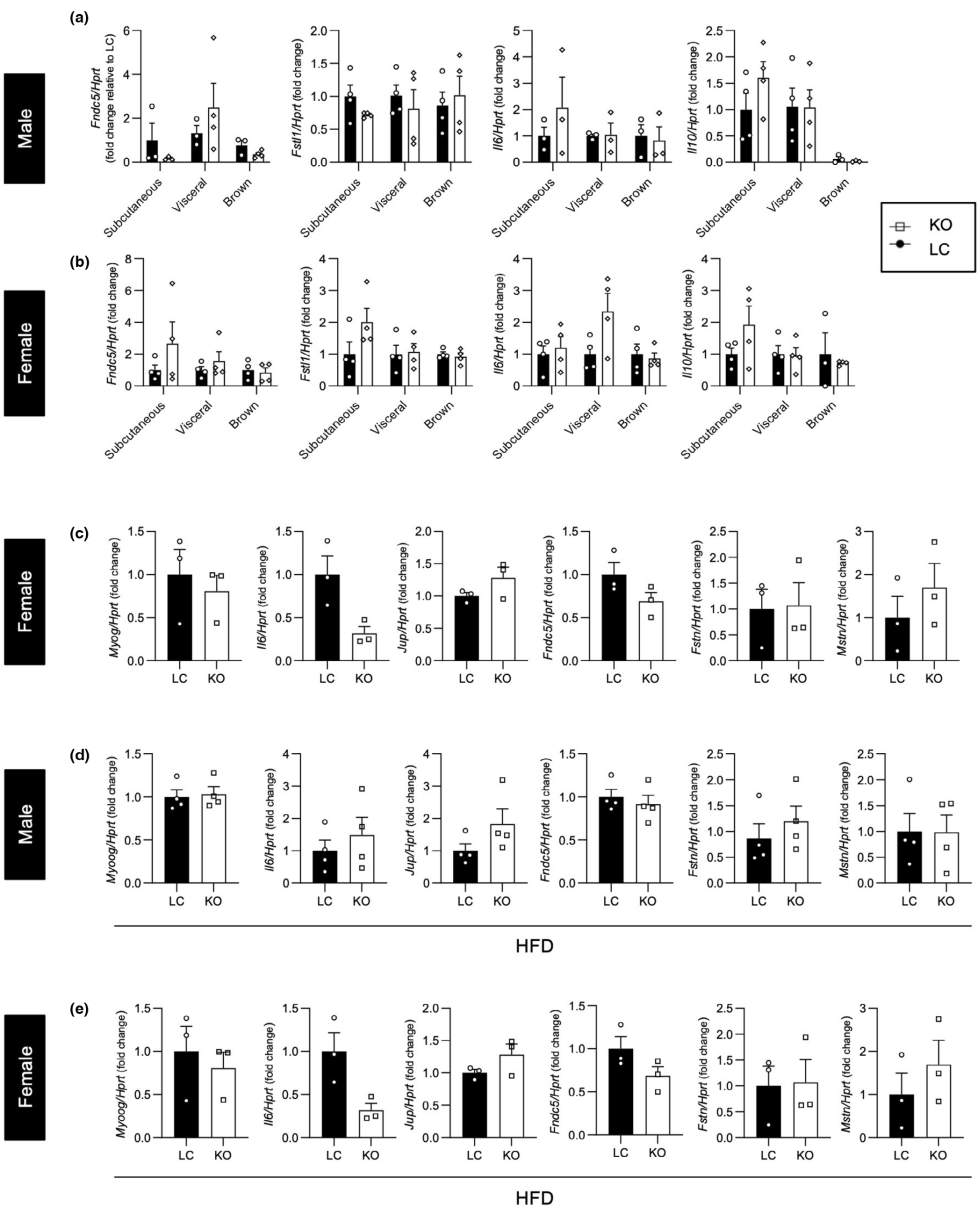
Genes promoting myogenesis are unaffected by adipocyte‐specific plakoglobin deletion (a, b) Expression profile of mRNA of adipo‐myokine genes in male (a) and female (b) subcutaneous, visceral and brown fat pads (*n* = 3–4 mice per group). (c–e) Expression profile of myogenic related genes in female skeletal muscle under chow (c), and high‐fat diet (d), and males under high‐fat diet (e) (*n* = 3–4 mice per group). Bar graphs represent mean ± SEM.

To potentially account for the differences in body weights seen in chow diet‐fed female mice (Figure 2j), we explored if there were differences in the expression of adipo‐myokines, which have been shown to regulate changes in adipocyte and skeletal muscle growth and metabolism (Li et al., 2017; Reza et al., 2017; Rotter et al., 2003; Wang et al., 2025; Weigert et al., 2005). In male or female KO mice, no differences were observed (Figure 8a,b).

We also assessed whether changes in the expression of genes underlying myogenesis or skeletal muscle hypertrophy were occurring. The levels of mRNA for *Myog* and *Mstn*, which encode key transcriptional regulators of myogenesis and skeletal muscle hypertrophy, respectively (Schuelke et al., 2004; Zammit, 2017), were not different in skeletal muscle under either diet condition (Figure 8c–e).

## 4 DISCUSSION

We previously identified plakoglobin as a regulator of adipogenesis in vitro and lipid accumulation in adipocytes. Its close homolog, β‐catenin, has previously been shown to inhibit adipogenesis in vitro and to protect against diet‐induced obesity when deleted within mature adipocytes in vivo. Research into plakoglobin's functions in primary, mature adipocytes has been limited, and in the present study, we now report that plakoglobin does not appear to influence adipogenesis in vivo. However, our study did reveal a sexual dimorphic role for adipocyte‐specific plakoglobin in the regulation of body weight. Challenging plakoglobin knockout mice with a 60% HFD further confirmed that plakoglobin does not influence adipogenesis or the expansion of adipose tissue mass. Worsened insulin resistance was observed only in HFD‐fed male mice, demonstrating another sexual dimorphic role of plakoglobin in adipocytes.

Systemic plakoglobin deletion was previously reported to result in embryonic lethality due to heart defects caused by the absence of desmosomes, and insights into its metabolic or developmental effects could not be determined (Ruiz et al., 1996). Murine models of arrhythmogenic right ventricular cardiomyopathy used an α‐myosin heavy chain (α‐MyHC) promoter to overexpress or delete plakoglobin (Lombardi et al., 2011), and the overexpression of plakoglobin led to its nuclear accumulation, inhibition of WNT transcriptional activity, and an increase in adipogenesis in cardiac myocytes (Lombardi et al., 2011). To our knowledge, our study is the first to investigate the specific roles of plakoglobin in mature adipocytes. Ablation of plakoglobin in adipocytes of female mice resulted in greater weight gain, without significant changes in lean or fat mass. Body weights of male KO mice under chow‐fed conditions were not different from littermate controls, consistent with effects observed when plakoglobin's homolog β‐catenin was deleted in mature adipocytes with *Ap2*‐cre or *Adipoq*‐Cre drivers (Chen et al., 2020; Zeve et al., 2012). This is at odds with studies performed in 3T3‐L1 cells, where both homologs were shown to regulate adipogenesis and affect lipid accumulation (Abou Azar et al., 2024; Cawthorn et al., 2007; Choi et al., 2020; Li et al., 2007). Lipolysis in response to an *i.p*. bolus of isoproterenol was relatively unaffected in male or female KO mice, despite differences in the abundance of HSL or mild depot‐specific differences that were observed between male and female mice.

Discrepancies between in vitro and in vivo models may arise from the fact that 3T3‐L1 cells are pre‐adipocytes and represent male murine cells that have already undergone the commitment stage of adipogenic differentiation, prior to the expression of adiponectin. The use of the *Adipoq*‐Cre promoter results in Cre recombinase being expressed in mature or differentiating adipocytes, representing a specific time point when plakoglobin may not have important contributions to differentiation (Farrar et al., 2021; Wang & Scherer, 2014). As an alternative, deletion of plakoglobin in adipocyte progenitor cells using a *Pdgfra*‐Cre driver may reveal additional functions of plakoglobin in the regulation of adipogenesis. The caveat being *Pdgfra* ^+^ cells are multipotent germline stem cells, which could lead to off‐target effects, as recombination can occur in fibroblasts, macrophages, and lymphocytes within adipose tissue (Berry & Rodeheffer, 2013; Chen et al., 2012; Kim et al., 2017; Krueger et al., 2014; Lee et al., 2012).

Unlike cardiac muscles, skeletal muscles lack desmosomes; however, plakoglobin has been shown to play a significant role in skeletal muscle growth and atrophy (Cohen et al., 2014; Eid Mutlak et al., 2020). Plakoglobin's presence was confirmed in satellite cells, as well as myoblasts, and downregulation of plakoglobin was reported to promote muscle atrophy (Cohen et al., 2014). Although observed changes in lean mass in female KO at the experimental endpoint were not statistically significant, our results motivated us to explore the potential of whether adipocyte‐specific deletion of plakoglobin could induce adipose–muscle cross‐talk to influence skeletal muscle mass. While no changes were observed in adipo–myokines or myogenic markers, further studies are required to elucidate a mechanism for patterns in lean mass increase, possibly by earlier time points, as effects on whole body weight waned at 23 weeks of age.

β‐catenin has been shown to regulate insulin signaling in adipocytes. For example, depletion in 3T3‐L1 adipocytes blunted insulin signaling, impairing the ability of GLUT4 to translocate to the plasma membrane (Dissanayake et al., 2018). However, ablation of β‐catenin in mature adipocytes, while improving glucose tolerance under high‐fat diet feeding, did not affect insulin sensitivity, highlighting the complexities of studying the catenin family of proteins (Bagchi et al., 2020). Plakoglobin has also previously been implicated in insulin signaling (Eid Mutlak et al., 2020; Negoita et al., 2020). Within muscle, it binds the insulin receptor, leading to increased activity of the PI3K‐AKT axis (Cohen et al., 2014), and depletion of plakoglobin was sufficient to impair insulin signaling and induce muscle atrophy (Eid Mutlak et al., 2020). In 3T3‐L1 adipocytes, knockdown of plakoglobin reduced PKB and AS160 phosphorylation and attenuated glucose uptake, an effect mediated by salt‐inducible kinase 2 (Negoita et al., 2020). In the present study, reduced insulin sensitivity was only observed in male KO fed a high‐fat diet, suggesting minor contributions of adipocyte‐specific plakoglobin to the regulation of whole‐body glucose homeostasis.

We recognize that a major limitation of our study is the low number of biological replicates in various experiments. The primary objective of assessing the adipocyte‐specific contributions of plakoglobin on adipogenesis was achieved, as *Jup* ablation did not result in differences in fat mass between male and female mice fed chow or high‐fat diet. Moreover, the masses of different fat pads did not differ between KO mice and their littermate controls, and our morphometric analyses showed no differences in the distribution of adipocyte size or overall adipocyte size following *Jup* ablation. Our use of littermate controls allowed us to control for any potential off‐target effects of Cre expression in adipocytes, giving us confidence in our results. Despite the low number of replicates, our collective findings led us to conclude that our results are not due to artifacts of statistical analyses.

In conclusion, we have expanded on our previous studies to further explore the in vivo contributions of plakoglobin in the regulation of adipogenesis. Ablation of plakoglobin in mature adipocytes did not affect adiposity or the expression of key adipogenic factors, but differences in body weight gain were observed, in a sex‐specific manner. In the context of high‐fat diet‐induced obesity, plakoglobin decreased insulin sensitivity, implicating a potential role in the regulation of glucose homeostasis. Additional studies are required to further delineate the exact mechanisms by which plakoglobin exerts its influence on weight gain and glucose homeostasis.

## AUTHOR CONTRIBUTIONS

FAA performed experiments, wrote the manuscript, and produced figures. AV, AIM, and FP performed analysis, performed experiments, produced figures, and edited the manuscript. MC performed analysis and edited the manuscript. WS provided reagents and edited the manuscript. GEL wrote and/or edited the manuscript. GEL is the guarantor of this work.

## CONFLICT OF INTEREST STATEMENT

The authors declare that they have no conflicts of interest with the contents of this article.

## ETHICS STATEMENT

All procedures were approved by the Comité institutionnel de protection des animaux du CRCHUM (CIPA, protocol CM25001GLs) and performed in accordance with CIPA guidelines at the Université de Montréal Hospital Research Centre (CRCHUM).

## Data Availability

The data that support the findings of this study are available from the corresponding author upon reasonable request.
